# Interaction of Silver-Lignin Nanoparticles With Mammalian Mimetic Membranes

**DOI:** 10.3389/fbioe.2020.00439

**Published:** 2020-05-08

**Authors:** Javier Hoyo, Kristina Ivanova, Juan Torrent-Burgues, Tzanko Tzanov

**Affiliations:** Grup de Biotecnologia Molecular i Industrial, Department of Chemical Engineering, Universitat Politècnica de Catalunya, Terrasa, Spain

**Keywords:** model mammalian membrane, silver-lignin nanoparticles, atomic force microscopy, quartz crystal microbalance, Langmuir film

## Abstract

Silver nanoparticles (AgNPs) have broad spectrum antibacterial activity, but their toxicity to human cells has raised concerns related to their use as disinfectants or coatings of medically relevant surfaces. To address this issue, NPs comprising intrinsically bactericidal and biocompatible biopolymer and Ag with high antibacterial efficacy against common pathogens and compatibility to human cells have been engineered. However, the reason for their lower toxicity compared to AgNPs has not yet been elucidated. This work studies the *in vitro* interaction of AgLNPs with model mammalian membranes through two approaches: (i) Langmuir films and (ii) supported planar bilayers studied by quartz crystal microbalance and atomic force spectroscopy. These approaches elucidate the interactions of AgLNPs with the model membranes indicating a prominent effect of the bioresourced lignin to facilitate the binding of AgLNPs to the mammalian membrane, without penetrating through it. This study opens a new avenue for engineering of hybrid antimicrobial biopolymer – Ag or other metal NPs with improved bactericidal effect whereas maintaining good biocompatibility.

## Introduction

Silver nanoparticles (AgNPs) possess many beneficial properties such antibacterial, antifungal, and antiviral activity (Bondarenko et al., 2013). They have been incorporated in clothing, cosmetics, and medical devices to control bacterial growth and reduce the infection occurrence (Rejeski, 2009). Despite their promising potential as antibacterial agents, effective also against antibiotic resistant bacteria, AgNPs are toxic and the release of silver ions (Ag^+^) from silver-containing products to the environment represents a global environmental concern (Bondarenko et al., 2013; Justo-Hanani and Dayan, 2014; Valsami-jones, 2015). Furthermore, the recovery or deactivation of those particles is difficult, time and money consuming (Walser et al., 2012).

Alternative approaches based on less toxic antibacterial metal-oxide NPs (Hoyo et al., 2019a), or biopolymers (Francesko et al., 2018; Ivanova et al., 2018) with inherent bactericidal activity have been proposed (Fernandes et al., 2017; Ivanova et al., 2017). However, most of these actives have shown lower antibacterial efficiency to drug resistant microorganisms when compared to AgNPs. To benefit from the outstanding performance of AgNPs in terms of microbial elimination, and at the same time reduce their toxicity and environmental impact, novel hybrid antimicrobial biopolymer-AgNPs have been engineered. The biopolymers replace the harsh reducing chemicals in AgNPs synthesis and form a biodegradable shell on the AgNPs core. This shell controls the silver ions release, reduces the toxicity to human cells and induces a synergistic antimicrobial effect (Richter et al., 2015).

Francesko et al. (2017) used chitosan, a natural biocompatible polymer with intrinsic bactericidal activity, as both reducing and capping agent in the production of AgNPs. Ferreres et al. (2018) developed NPs of amylase, an enzyme with antibiofilm properties that simultaneously reduced and capped AgNPs. Richter et al. (2015) and Nix et al. (2018) reported the synthesis of lignin NPs infused with Ag^+^and coated with cationic polyelectrolyte. The hybrid NPs showed strong antimicrobial activity against a number of Gram-positive and -negative pathogens at 10-fold lower concentration of Ag than the conventional AgNPs (Beisl et al., 2017). In contrast to AgNPs alone, such biopolymer-silver nanocomposites have lower environmental impact because of the rapid Ag^+^ depletion upon utilization and the consequent biodegradation of the remaining lignin core after disposal (Richter et al., 2015). In the aforementioned examples, the presence of biocompatible and intrinsically antibacterial polymers provided bactericidal efficacy of the nanocomposites at lower silver content and reduced their toxicity to human cells. Despite their relevance for medical purposes, the mechanism of interaction of such biopolymer-AgNPs with mammalian cells still remains largely unexplored.

The current work is a mechanistic study *in vitro* of the interaction of AgLNPs with two mammalian membrane models aiming to elucidate the reason for the low toxicity of these particles. Lignin is a non-toxic (Vinardell and Mitjans, 2017) natural phenolic polymer (Frangville et al., 2012; Beisl et al., 2017) with reported antimicrobial activity (Cazacu et al., 2013), which additionally possesses multiple functional groups that can reduce Ag^+^ for the synthesis of AgNPs (Hu and Hsieh, 2016).

Cell membrane models of mammalian (Domènech et al., 2006), thylakoid (Hoyo et al., 2012, 2015, 2016), and bacterial (Michel et al., 2017; Hoyo et al., 2019b) membranes using Langmuir monolayers have been used to study molecular level interactions. This technique allows the membrane-like organization of lipids to assess the intermolecular forces that actives, e.g., antimicrobial NPs, present in the subphase exert on the membrane. The interaction of bioactive polymers such as chitosan and hyaluronic acid with model mammalian membranes has been previously studied (Pasquali-ronchetti et al., 1997; Pavinatto et al., 2016). On the other hand, quartz crystal microbalance with dissipation (QCM-D) has also been used to analyze molecular protein–protein (McMillan et al., 2013), bacteria–NPs (Ferreres et al., 2018), and lipid–peptide (Shahmiri et al., 2015) interactions providing a real-time output. Herein, two kinds of mammalian mimetic membranes (i) Langmuir films at the air–water interface and (ii) supported lipid bilayers (SLB) established on the QCM-D sensor, were exposed to AgLNPs. The SLB were further analyzed by atomic force microscopy (AFM). These analytical techniques provided information about the mechanism of action of AgLNPs on mammalian cells highlighting the improved biocompatibility of the lignin-capped AgNPs.

## Materials and Methods

### Materials

Lignin (alkali-low sulfonate), silver nitrate (AgNO_3_) and 1,2-dimyristoyl-sn-glycero-3-phosphocholine (DMPC) were obtained from Sigma-Aldrich (Spain). All other reagents used for QCM-D disks functionalization and cleaning, or buffers and subphase preparation were obtained from Sigma-Aldrich (Spain). Ultrapure MilliQ water with a resistivity of 18.2 MΩ cm was used in the experiments.

### Synthesis of AgLNPs

Water-based lignin solution (1% w/v) was prepared and the pH was adjusted to 5.5 with HCl (5 M). Then, 20 mL of 2 mg mL^–1^ aqueous AgNO_3_ solution were added to 30 mL of 1% lignin solution and the reaction was carried out in a glass vessel for 3 days at 60°C under continuous stirring (250 rpm). After the reaction, the excess of silver salt was removed by centrifugation at 18,000 g for 40 min. The AgLNPs were resuspended in 5 ml MilliQ water and subjected to further analysis.

### NPs Characterization

The formation of AgLNPs was confirmed by UV–VIS spectroscopy in the range of 300–600 nm using an Infinite M200 spectrophotometer (TECAN). Nanoparticle tracking analysis (NTA, NanoSight NS 300 – Malvern Instruments Inc., United Kingdom) in flow mode and software NTA 3.2 were used to obtain the hydrodynamic diameter and final concentration of the particles. The morphology and size of the AgLNPs were determined using TEM (JEOL JEM-2100 LaB6) operating at an accelerating voltage of 200 kV using SiO_2_ grid. Energy-dispersive X-ray spectroscopy (EDX) chemical maps were acquired using STEM mode with a High Angle Annular Dark Field (HAADF) detector to reveal the chemical composition of the AgLNPs. The used spectrometer is an Oxford Instruments INCA x-sight, with Si (Li) detector and maps were acquired using the INCA Microanalysis Suite version 4.09 software. The ζ-potential of AgLNPs was measured using a Zetasizer Nano ZS (Malvern Instruments Inc., United Kingdom).

### DMPC Liposomes Preparation

The DMPC solution (1 mg mL^–1^) was dried in a rounded bottom tube under gentle and slow nitrogen flow and dried overnight. Afterward, lipids were resuspended in buffer solution maintaining the same lipid concentration. Six cycles of 50 s vortexing and heating (60°C) followed by 30 min sonicating (30°C) were performed to obtain unilamellar vesicles (Hoyo et al., 2013) 100 mM NaCl and 20 mM K_2_HPO_4_ aqueous buffer solution pH 6.95 was used for preparing liposomes, Langmuir subphase and QCM-D cleaning solutions.

### Interaction of the NPs With Mammalian Membrane Models

The interaction of AgLNPs with the mammalian membrane model at the air–liquid interface was evaluated by the use of Langmuir films. Monolayers of DMPC, AgLNPs, and the corresponding NPs individual components were formed in a Langmuir trough (KSV NIMA Langmuir–Blodgett Deposition Troughs, model KN2002, Finland) equipped with two mobile barriers mounted on an antivibration table, housed in an insulation box at 23 ± 1°C. The surface pressure (π) was measured by a Wilhelmy balance connected to the trough. The Langmuir trough was cleaned with chloroform and water several times and the control isotherm of the pristine subphase confirmed the cleanliness. All the experiments were carried out at least three times with barrier closing rates at 15 cm^2^ min^–1^.

π-Area (π-A) isotherms were prepared by pouring the subphase (buffer – section “DMPC Liposomes Preparation” – in which the soluble compounds are dissolved) into the through and adding 25 μL of DMPC. The π-A isotherm recording started after 10 min of lagging for complete chloroform evaporation.

The physical states of the monolayers were estimated by the inverse of the compressibility modulus $C_{\text{s}}^{-1}$ that is obtained from the π-A isotherms calculated according to Eq. 1, where *A* is the mean area per molecule (Å^2^ molecule^–1^), π the surface pressure (mN m^–1^) and *T* the absolute temperature (K).

(1) $$C_{\text{s}}^{-1}=-A{\left(\frac{d\pi }{dA}\right)}_{\text{T}}$$

Quartz crystal microbalance (QCM-D, E4 system, Q-Sense, Sweden) was used to analyze *in situ* the effect of the AgLNPs on a mammalian mimetic bilayer. Gold sensors (QSX 301, QSense, Sweden) were cleaned in a ultrasound bath successively with acetone, ethanol and isopropanol for 10 min at 40°C. Afterward, the disks were dried under nitrogen and treated with 2% (w/w) 3-mercaptopropionic acid (MPA) in isopropanol overnight to form a self-assembled monolayer (SAM) that facilitates the formation of a biomimetic membrane on the disk surface (Shahmiri et al., 2015). Thereafter, isopropanol was used to rinse the QCM-D disks for removing loosely attached MPA molecules. The disk was dried under nitrogen stream and placed in the QCM-D flow cell at 37°C. The abovementioned buffer (section “DMPC Liposomes Preparation”) was circulated until stabilization of the system. All reagents, cleaning and surfactant (5% w/w of Poloxamer 407 in buffer) solutions were flushed at 20 μL/min. To simplify the data interpretation, only the normalized frequency (Δ*f*/ν) and dissipation (Δ*D*) shifts as a function of time of one representative sample per experimental group (5th harmonic) are shown.

### Surface Characterization of Mammalian Cell Membrane Models

Atomic force microscopy topographic images of the films formed on the gold sensors were acquired in an air tapping mode using a Multimode AFM controlled by Nanoscope IV electronics (Veeco, Santa Barbara, CA, United States) under ambient conditions. The gold sensors were glued to the AFM holders using and adhesive paste (Nural 27, Pattex) to reduce the influence of the sensors irregular bottom surface. Rectangular AFM probes with antimony (n) doped silicon cantilevers were used (RTESPA-150, Bruker) with a nominal spring constant of 5 N m^–1^ and a resonant frequency of 150 kHz. Images were acquired at 1 Hz line frequency and at minimum vertical force to reduce sample damage. Images were processed using Nanoscope Analysis 1.8 software.

## Results and Discussion

### AgLNPs Characterization

**FIGURE 1:**
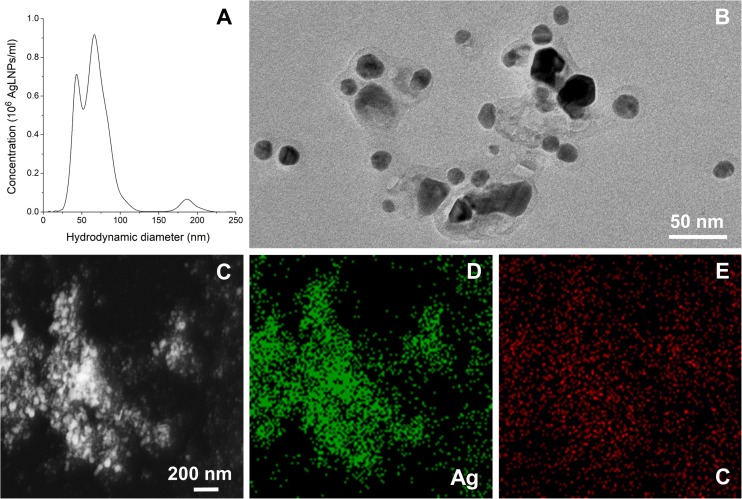
**(A)** Hydrodynamic diameter distribution of the AgLNPs. **(B)** TEM image of the AgLNPs. **(C)** TEM image of AgLNPs using the HAADF detector and EDX, showing Ag **(D)** and carbon **(E)** mapping.

Hybrid AgLNPs were synthesized in one-pot reaction using lignin as both reducing and capping agent. The formation of AgLNPs was confirmed with UV-vis spectrophotometry by the appearance of a peak for Ag^0^ at λ ≈420 nm (Hu and Hsieh, 2016; Supplementary Figure S1). The synthesis of AgLNPs yielded two main NPs populations with hydrodynamic diameter of 44 and 67 nm, and a residual population of 187 nm (Figure 1A). The lower polydispersity and the negative ζ-potential of -41.03 ± 0.91 mV confirmed the stability of these spherical in shape AgLNPs.

TEM image of the AgLNPs showed a core-shell morphology, with a dark center and a brighter halo corresponding to Ag^0^ and lignin, respectively (Figure 1B). Furthermore, the HAADF scanning TEM (STEM) mapping and the EDX elemental maps of the corresponding TEM image revealed that silver was mainly present in the core of the particles (Figures 1C,D), while lignin was forming a thin polymeric shell (Figures 1C,E). The carbon map of the nanocomposites (Figure 1E) showed a nearly homogeneous distribution of carbon (red color), although a higher carbon content was observed in the zones with higher Ag concentration (Figure 1D, green color) indicating the formation of hybrid AgLNPs. Moreover, the similar ζ-potential of the AgLNPs and lignin in free form (−35.9 ± 1.5 mV) pointed out the presence of lignin in the external layer of the particles. The high negative potential of the AgLNPs was mainly due to the phenolic and carboxyl groups of the lignin shell and partially resulted from the adsorption of hydroxyl ions from water on the NPs’ surface (Frangville et al., 2012; Lievonen et al., 2016).

### Interaction of AgLNPs With Mammalian Model Membrane

**FIGURE 2:**
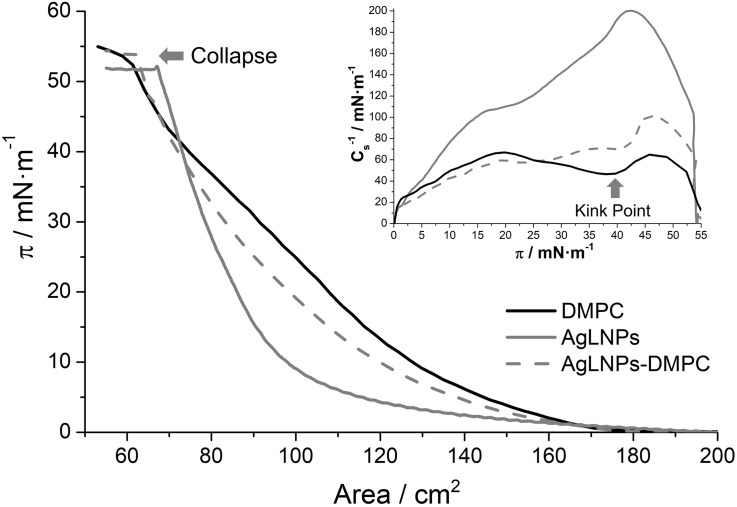
π-A isotherm in buffer of a DMPC, AgLNPs and AgLNPs-DMPC monolayer and the respective inverse of the compressibility modulus (Inset) (*n* = 3).

**FIGURE 3:**
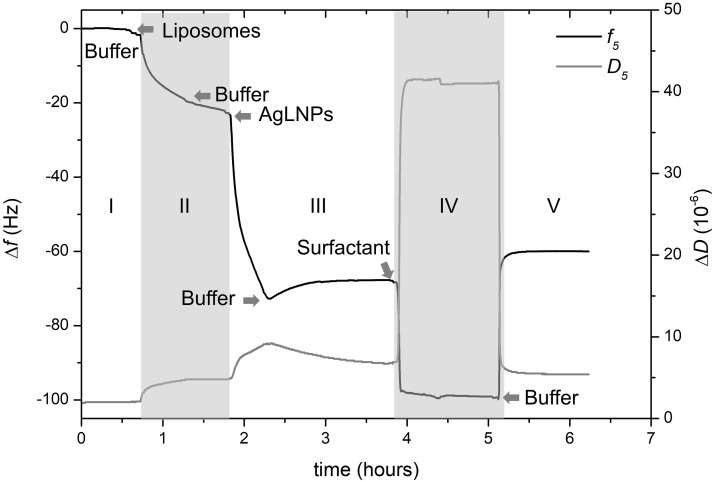
QCM-D monitoring of normalized frequency (Δ*f*_5_) and dissipation (Δ*D*_5_) obtained during the formation of the model biomembrane and its interaction with AgLNPs. Arrows indicate the circulation of a new fluid (*n* = 3).

The interaction of AgLNPs with mammalian membranes was assessed by means of Langmuir films (Figure 2) and QCM-D (Figure 3) using DMPC lipid as a model for mimicking mammalian membranes (Hall et al., 2014; Marquardt et al., 2015; Shahmiri et al., 2015). Monolayers of DMPC alone or in combination with AgLNPs and the corresponding NPs individual components were prepared to evaluate the DMPC–AgLNPs interactions and the surface activity of the NP constituents. Langmuir films have been used to study *in vitro* the binding and/or penetration of molecules through model mammalian membranes, in particular for systems affecting the elasticity of the membrane (Pavinatto et al., 2007; De Brito et al., 2011).

In accordance with previous studies, the DMPC π-A isotherm increases monotonically until the collapse pressure at 54 mN m^–1^ (Miyoshi and Kato, 2015). The fluidity of the DMPC films reached a maximum value in the inverse of the compressibility modulus $C_{s\max}^{-1}$ of 65 mN m^–1^ (Inset of Figure 2) that correlates with a liquid expanded (LE) state (Vitovič et al., 2006). The kink point observed at ≈40 mN m^–1^ may correspond to reorientation of the lipid molecules in the LE state. On the other hand, the AgLNPs π-A isotherm demonstrated a monotonic increase until the collapse at 52 mN m^–1^ with absence of kink points in the $C_{\text{s}}^{-1}$curve. The $C_{s\max}^{-1}$≈200 mN m^–1^ indicated the LC state (Vitovič et al., 2006) of the monolayer. The interaction of both compounds (AgLNPs-DMPC curve) yielded a π-A isotherm positioned between the isotherms of AgLNPs and DMPC, respectively. As previously observed for π-A isotherms of lipid mixtures (Hoyo et al., 2016), the most fluid compound leads the fluidity of the mixture as demonstrated by the similarity of the DMPC and AgLNPs–DMPC $C_{\text{s}}^{-1}$ curves. The minor differences between these curves indicated a negligible interaction between AgLNPs and DMPC film. The AgLNPs–DMPC isotherm had slightly lower surface area than the isotherm of pure DMPC, which demonstrated that AgLNPs have a minor effect in favoring the ordering of the DMPC molecules in the monolayer. Negligible coupling effects of polyelectrolytes to monolayers of zwitterionic lipids have been reported for dipalmitoylphosphocholine (Bordi et al., 2003) and dipalmitoylphosphatidylethanolamine (de Meijere et al., 1998). In our case, the most probable explanation is that the negatively charged AgLNPs partially screen the electrostatic repulsion between the zwitterionic DMPC headgroups (a negative charge on the phosphate group and a positive charge on the quaternary ammonium group) causing denser packing and stronger hydrophobic interaction between the lipid chains, thus stabilizing the LE phase (Krajewska et al., 2013).

The π-A isotherms of the pristine counterparts of AgLNPs and their interaction with DMPC were also studied (Supplementary Figure S2). The surface activity of AgLNPs could be assigned to their lignin capping (Supplementary Figure S2), since a lack of surface activity of metallic NPs without stabilizer was previously reported (Constantino et al., 1996; Barros et al., 1999). In this sense, the interaction of AgNPs with DMPC was negligible, while the interaction of lignin with DMPC resembled that of the AgLNPs. The shape of the AgLNPs isotherm was similar to that of the bulk lignin, although the isotherm of lignin started at larger surface area. Such difference was probably due to incomplete conversion of lignin into AgLNPs. Lignin and AgLNPs slightly altered the stability and compactness of the model membrane (Supplementary Figure S2). AgLNPs minor interaction with the DMPC polar heads points that AgLNPs are in contact with the membrane but they are not capable of penetrating it. The QCM-D and AFM analysis will confirm that AgLNPs remain attached on the surface of the mammalian membrane model, being the reason for the reduced mammalian cell damage.

Quartz crystal microbalance with dissipation experiments were further carried out to evaluate *in situ* the interaction of AgLNPs with model mammalian membranes (Indest et al., 2009). A biomimetic membrane based on a SLB of DMPC was prepared on the gold surface of QCM-D disks following already described SLB models (Jing et al., 2014; Shoaib et al., 2017). The surface was functionalized with MPA to allow the formation of a thiol SAM with hydrophilic carboxyl groups that will enhance the deposition and then the opening of previously formed DMPC liposomes. Opening the liposomes resulted in the formation of a membrane-mimicking lipid layer on the gold QCM-D disks and allowed for *in situ* evaluation of its interaction with AgLNPs (Figure 3).

**FIGURE 4:**
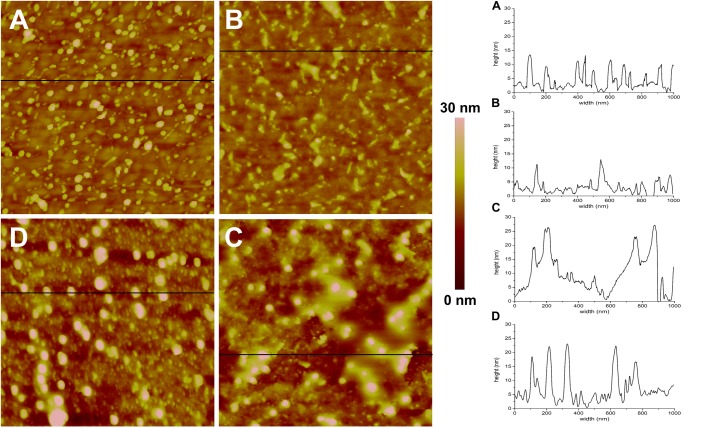
AFM topographic images (1 × 1 μm) and representative profiles of the QCM-D gold disks surface. **(A)** Functionalized with MPA, **(B)** MPA functionalized with DMPC, **(C)** MPA functionalized with DMPC after application of AgLNPs, **(D)** MPA functionalized with DMPC and AgLNPs, after surfactant circulation (*n* = 3).

After establishing the first baseline in buffer (Figure 3, zone I), a suspension of DMPC liposomes was flushed through the system to deposit the liposomes on the carboxy-functionalized gold surface. Then a cleaning buffer for removal of the unopened liposomes was circulated (Figure 3, zone II). This step permitted the formation of the lipid layer and established the second baseline. Once the biomimetic membrane was formed on the QCM-D surface, AgLNPs were circulated and finally buffer was used to set the third baseline (Figure 3, zone III). Surfactant was also flushed to assess the binding of AgLNPs on the lipid layer leading to the establishment of the fourth baseline (Figure 3, zone IV) prior to circulating a buffer to draw the fifth baseline (Figure 3, zone V). QCM-D fingerprinting, i.e., plotting of the QCM dissipation versus the frequency change (Δ*D* vs. Δ*f*) of the system (Supplementary Figure S3), was analyzed. AFM images and representative height profiles were taken in each baseline to characterize the surface variation after each step (Figure 4).

The magnitude and shape of the frequency decrease in zone II (Figure 3) indicated the liposomes deposition on the functionalized QCM-D disk, their rupture and fusion, leading to a lipid bilayer formation (Keller and Kasemo, 1998) also confirmed by the topographical images (Figure 4B). The negative shift of 25 Hz was assumed to correspond to a single bilayer (Mechler et al., 2009), which correlates well with the values obtained herein. The negative Δ*f* combined with positive Δ*D* (Supplementary Figure S3) means mass uptake (Shahmiri et al., 2015), which confirmed the deposition of a viscoelastic (not rigid) film on the functionalized disk. Once the adsorption of the bilayer was completed, the dissipation remained invariable.

The circulation of AgLNPs induced a sharp negative shift in the frequency that was not recovered after buffer circulation (zone III of Figure 3). The QCM-D fingerprinting (Supplementary Figure S3) showed a large negative frequency change coupled to a relatively low dissipation increase, which suggested that a tightly coupled layer was formed on the surface. This result correlates well with an initial surface binding of the AgLNPs onto the film and the partial removal of the loosely adhered NPs by the circulating buffer [Figure 4C (bright circles)].

A surfactant was circulated through the system to test the binding of AgLNPs to the SLB. The sharp changes in frequency and dissipation observed in the QCM-D measurements (Figure 3 and Supplementary Figure S3) immediately after the addition of the surfactant solution could be related to the so called “bulk effect” (Tammelin et al., 2006) due to the different properties of the surfactant solution, rather than to a change in the membrane properties. Minor positive frequency and dissipation shifts (zone V of Figure 3) were produced by circulating the rinsing buffer. The surfactant washed away only a negligible amount of loosely bound to the monolayer AgLNPs as was further evidenced by the presence of high quantity of AgLNPs (bright circles) in the AFM topographic images (Figure 4D).

### AgLNPs Effect on Model Mammalian Membranes

The use of lignin in the AgLNPs synthesis has two main advantages: (i) lignin acts as both reducing and capping agent, replacing the harsh reducing and stabilizing chemicals in the NPs production and (ii) lignin-containing silver NPs showed enhanced antibacterial efficacy at lower amounts of Ag coupled to improved biocompatibility with human cells (Richter et al., 2015; Nix et al., 2018).

However, the mechanism of action of these biocide particles against bacterial and mammalian membranes is not fully understood and seems different. It is possible that the AgLNPs adsorb on bacteria cells, creating increased local concentration on the surface, from where they progressively release Ag^+^, leading to membrane disturbance and cells death. In contrast, this study demonstrated that AgLNPs adhered to the DMPC lipids (Figures 3,4) found in mammalian cells (different in structure from the lipids in bacterial membranes), without disturbing significantly the mammalian model membrane (Figure 2) and therefore would not be toxic to human cells as reported elsewehere (Richter et al., 2015; Nix et al., 2018). Non-specific SLB-NPs interactions have been demonstrated to affect the structure and elasticity of the lipid bilayer, causing physical state changes, formation of lipid domains or lipid bilayer disruption (Di Silvio et al., 2017). The surface activity of AgLNPs was due to the presence of a lignin shell in their structure (Figure 1A and Supplementary Figure S2). The interactions of AgLNPs with mammalian mimetic membranes in aqueous phase (Figure 2) could be, therefore, based on electrostatic forces between the phenolic and carboxyl groups of the biopolymer and the phospholipid polar heads from the membrane, combined with van der Walls forces, hydrophobic and hydrogen bonding. These forces contribute both to the distortion of the phospholipid tails and the conformational changes of the biopolymer in solution (Pavinatto et al., 2007; Fernandes et al., 2013). In our study, the mammalian model membrane was prepared using DMPC, a lipid with a zwitterionic headgroup, which induces low electrostatic interaction with the negatively charged surface of the AgLNPs. Therefore, the weak non-specific interactions described above increase their relevance. Lignin is a hydrophilic compound able to form strong inter- and intramolecular hydrogen bonds due to the presence of a large number of hydroxyl groups (Pavinatto et al., 2014; Wang et al., 2015). The hydrophilicity of the AgLNPs shell shields them from the phospholipid tails, thus avoiding their penetration into the mammalian membrane, as observed in Figure 4 and inferred from Figure 2.

## Conclusion

In this work, lignin was used as both reducing and capping agent in the one-pot green synthesis of AgLNPs. *In vitro* assessment of the interactions of AgLNPs with Langmuir films and SPB mammalian membrane models confirmed that the lignin shell of the particles was responsible for their binding onto the mammalian cell surface. The negatively charged AgLNPs partially compensated the electrostatic repulsion of the zwitterionic headgroups of mammalian lipids, resulting in a denser packing and stronger interaction of the hydrophobic chains. Additionally, the hydrophilic nature of lignin could reduce the AgLNPs interaction with the phospholipid tails in the membrane, thus avoiding the membrane permeability of the antimicrobial nanocomposites minimizing their cellular toxicity.

## Data Availability Statement

The datasets generated for this study are available on request to the corresponding author.

## Author Contributions

JH and TT contributed the conception and design of the study. JH and KI were involved in the experimental section. All authors were involved in the results discussion and contributed to manuscript revision, read and approved the submitted version.

## Conflict of Interest

The authors declare that the research was conducted in the absence of any commercial or financial relationships that could be construed as a potential conflict of interest.
