# Supplementary material for: Interaction of Silver-Lignin Nanoparticles With Mammalian Mimetic Membranes
Source: Front Bioeng Biotechnol. 2020 May 8;8:439. doi: 10.3389/fbioe.2020.00439 (PMC7225684; doi:10.3389/fbioe.2020.00439)
Supplement: Supplementary file 1 [file Table_1.DOCX]

**Supporting Information**


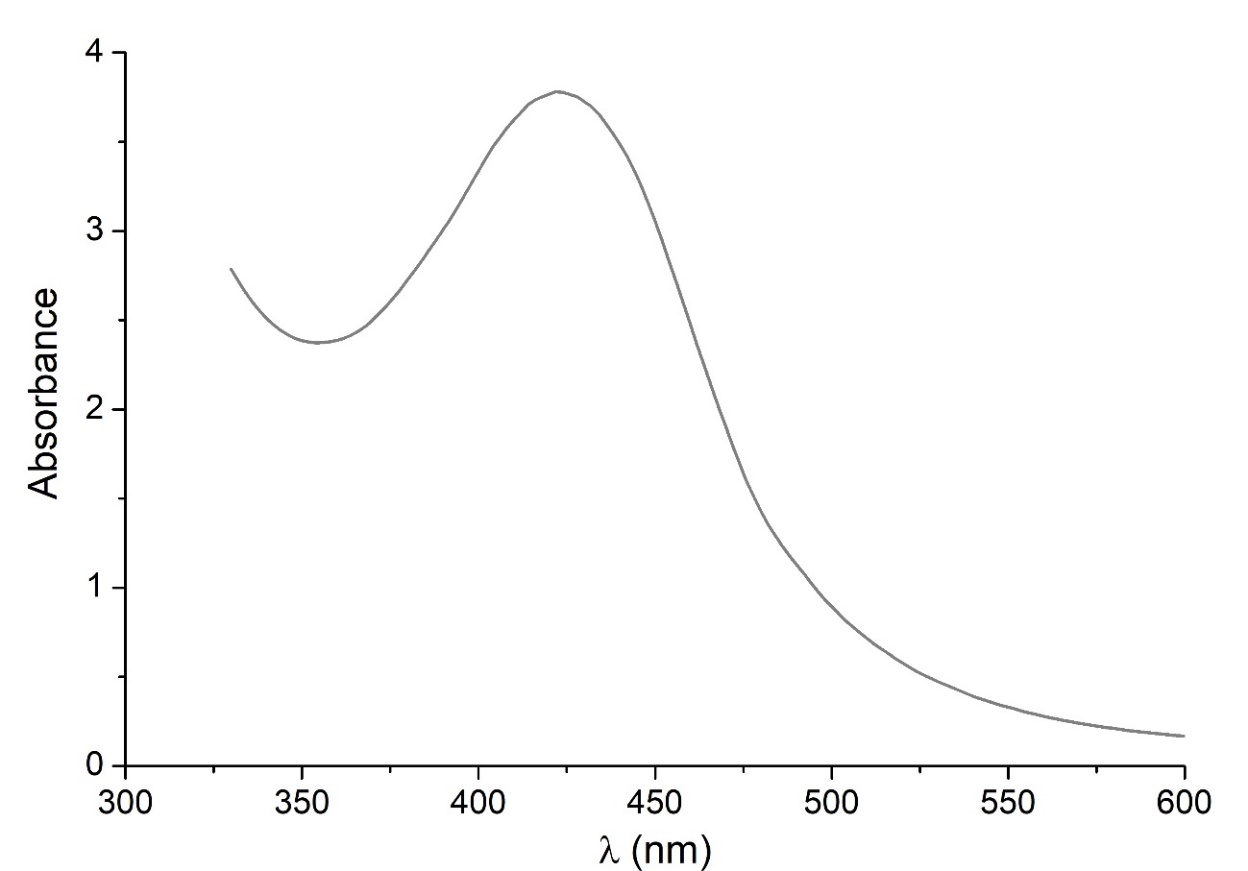


**Figure S1.** UV–vis spectra of AgLNPs


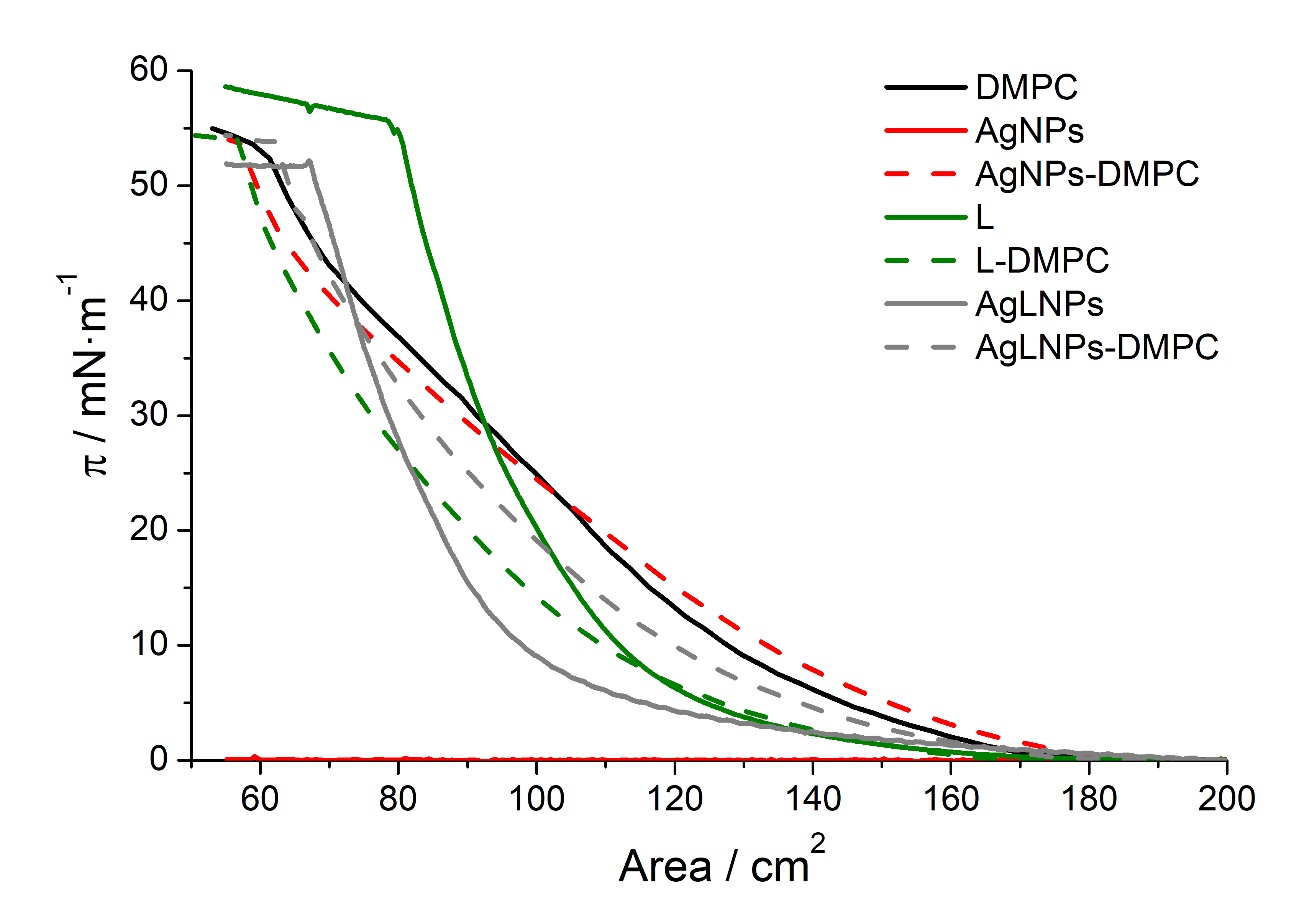


**Figure S2.** Surface pressure-area isotherm in buffer of monolayers of AgLNPs, their pristine compounds and their interaction with DMPC.
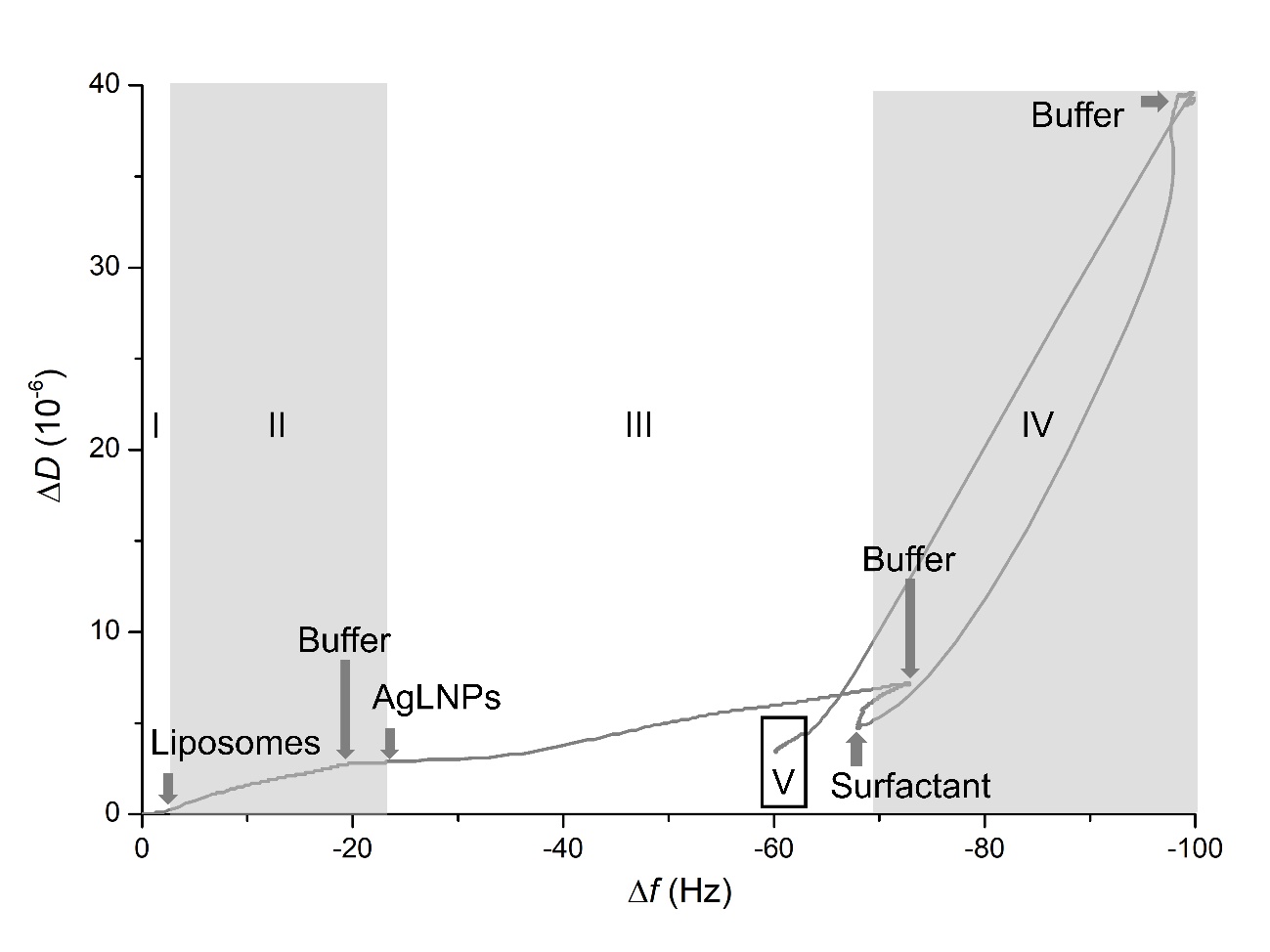


**Figure S3.** Plot of Δ*D*_5_-Δ*f*_5_ -sensorgram presented in Fig 3- obtained during the formation of the model biomembrane and its interaction with AgLNPs. Arrows indicate the circulation of a new fluid.
